# Multimodal neuroimaging data boosts the prediction of multifaceted cognition

**DOI:** 10.21203/rs.3.rs-7721822/v1

**Published:** 2025-11-19

**Authors:** Jianxiao Wu, Jingwei Li, Kyesam Jung, Simon Eickhoff, B. T. Thomas Yeo, Sarah Genon

**Affiliations:** Heinrich Heine University Düsseldorf; Heinrich Heine University Düsseldorf; Research Centre Jülich; Research Center Juelich; National University of Singapore; Research Centre Jülich

## Abstract

Relating individual brain patterns to behavioural phenotypes through predictive modelling has been increasingly popular. Several recent studies have focused on the fundamental challenge of improving behavioural prediction based on individual brain patterns, by integrating information from multimodal neuroimaging data. However, the benefit of multimodal integration in brain-based behaviour prediction remains debated due to inconsistent findings. This issue raises the need of a systematic and extensive evaluation. Here, we investigated the necessity and benefit of multimodal integration in 3 large datasets covering different age ranges, using 25 to 33 feature types from different imaging modalities, and 21 behavioural measures from different domains. By setting up multiple predictive models corresponding to increasing levels of multimodal integration, we demonstrated that prediction performance saturates after integrating a few types of features. In general, our analyses revealed that multifaceted cognitive scores tend to require higher levels of multimodal integration, while other predictions may depend on single feature types. In most cases, multimodal integration can remain focused on functional features, especially in young adults. However, predictions in aging can also require structural and diffusion features. Along the same line, while model-free rest and task functional connectivity may provide relevant brain phenotype for behavioural prediction in most applications, in aging, effective connectivity appears relevant too. Thus, our study demonstrates that alternatives to model-free functional connectivity and, more generally, to functional imaging features should be considered for predictive modelling of behaviour, especially in aging populations where understanding interindividual variability in remain as a key challenge.

## Introduction

The study of the relationships between individual differences in brain phenotypes and individual behaviours is fundamental in neuroscience. In recent years, the study of brain-behaviour relationships through predictive modelling, or brain-based psychometric prediction, has grown into a prominent field (Sui et al., 2020; Yeung et al., 2022; Wu et al., 2023). The general approach involves training a machine learning model to predict particular behavioural variables from brain-based data from a number of individuals (the training set), and evaluating its performance on unseen data (the test set). At the forefront of the field of brain-based psychometric prediction, researchers have identified and been attempting to resolve the challenges of low prediction accuracies, limited generalizability, and difficulty in interpretation (O’Connor et al., 2021; Rasero et al., 2021; Wu et al., 2021; Marek et al., 2022; Ooi et al., 2022; Pat et al., 2022; Rosenberg et al., 2022; Wu et al., 2022; Rauland et al., 2025; Tetereva et al., 2025). Most urgently, the current low range of prediction accuracies is a bottleneck that requires addressing before other challenges could be meaningfully discussed. A commonly investigated solution is to integrate information from multimodal neuroimaging features to boost prediction performance (Jiang et al., 2020a; Rasero et al., 2021; Ooi et al., 2022; Pat et al., 2022; Schultz et al., 2024; Tetereva et al., 2025). In particular, different Magnetic Resonance Imaging (MRI) modalities, such as structural, functional, and diffusion, probe different, albeit complementary, neurobiological aspects and hence could be combined to increase prediction accuracy.

While several studies have assessed the benefit of multimodal integration in terms of prediction performance, there has been no consensus on whether this approach improves prediction performance compared to a single-modality approach, nor what modalities and types of features may be useful for a multimodal approach. Many studies have shown that combining task and resting-state functional MRI boosts prediction performance (Gao et al., 2019; Jiang et al., 2020b; Chen et al., 2022). On the other hand, studies reported diverging results on whether integrating diffusion MRI features with functional MRI features improves prediction accuracies (Rasero et al., 2021; Ooi et al., 2021). Furthermore, some studies reported no improvement in prediction accuracies when integrating multimodal MRI data (Dhamala et al., 2021; Xiao et al., 2021). These disagreements could stem from many sources of methodological considerations, such as differences in phenotype choice or processing, MRI data preprocessing, feature computation, and cohort composition. As a result, comparison of results across studies is not straightforward.

**Figure 1:**
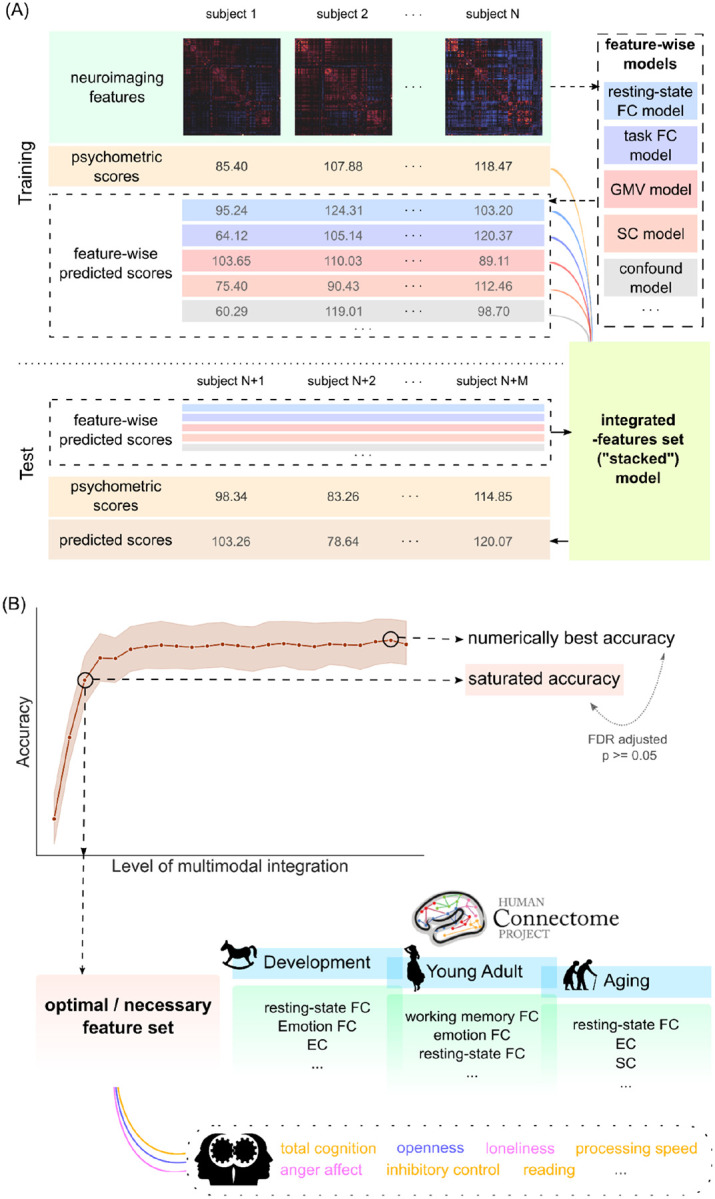
(A) Overview of the stacking procedure, within one cross-validation split. (B) Overview of study design

To this end, we conducted a systematic evaluation of multimodal psychometric prediction across multiple cohorts spanning a large age range, with an extensive set of neuroimaging features and psychometric measures (see an overview in Fig. 1). We used three datasets with a large set of overlapping psychometric variables and high-quality multimodal neuroimaging data, the Human Connectome Project Young Adult (HCP-YA; Van Essen et al., 2013), the Human Connectome Project Aging (HCP-A; Harms et al., 2018; Bookheimer et al., 2019), and the Human Connectome Project Development (HCP-D; Harms et a., 2018; Somerville et al., 2018). Overall, we selected 21 psychometric variables from cognition, emotion, and personality domains, as well as 25 to 33 types of neuroimaging features from resting-state functional MRI (rs-fMRI), task functional MRI (t-fMRI), structural MRI (sMRI), and diffusion MRI (dMRI). Building on the popular stacking procedure, we implemented multiple integrated-features set models (i.e., “stacked” models) each reflecting incremental levels of multimodal neuroimaging feature integration. By statistically comparing the prediction performance of these integrated-features set models, we can objectively deduce the need for multimodal integration and the type of features required for each psychometric measure in each cohort.

We first assessed the trend of prediction performance as more types of features were added to the integrated-features set models, showing that prediction accuracies saturate quickly with only a few types of features in most cases. Then, we examined the types of features used in the integrate-features set models with sufficiently saturated prediction performance (i.e., the “necessary” features). In general, we demonstrated that the need for multimodal neuroimaging feature integration is dependent on both the target behavioural variable and the population of interest. Multifaceted cognitive scores, specifically, call for higher levels of multimodal integration. Our analysis demonstrated that, while resting-state and task model-free functional connectivity features are predominantly predictive as suggested by existing studies, grey and white matter feature types are often required too. In particular, our results highlighted the importance of some less commonly discussed features in the aging cohort, such as cortical surface area, structural connectome, and dynamic causal modelling based effective connectivity, suggesting that explaining interindividual variability in cognitive aging requires multimodal approach including directional connectivity. Finally, we identified the useful feature types for well predicted psychometric variables as reference for future studies.

## Results

### Prediction Performance Improvement with Multimodal Integration.

We made use of data from 3 large cohorts, HCP-YA (N = 796), HCP-A (N = 700), and HCP-D (N = 593). For each dataset, 25–33 types of neuroimaging features were extracted from 4 imaging modalities (rs-fMRI, t-fMRI, sMRI, and dMRI), and 21 psychometric variables were selected as prediction targets from 3 domains (cognition, emotion, and personality). We included commonly used neuroimaging features such as resting-state and task model-free functional connectivity (FC), morphometry measures, diffusion tensor imaging features, and structural connectome, as well as less commonly considered features such as dynamic causal modelling based effective connectivity and morphometry measure based connectivity using structural coregistration. Prediction models were trained and tested using elastic net in a nested cross-validation scheme, where hyperparameters were estimated using the inner training data. A stacking procedure was implemented to integrate different neuroimaging features. Multiple integrated-features set (i.e., meta-level) models were built using Random Forest regression with increasing number of top feature types, corresponding to incremental levels of multimodal integration. To avoid data and information leakage, ranking of feature types were determined based on training set accuracy in each cross-validation split. Any parameter required for neuroimaging features based on a group model (e.g., for functional gradient) was computed only using training subjects and applied to the test set in each split. Furthermore, all input features to the integrated-features set (i.e. meta-level) model were also computed in a nested cross-validation to prevent leakage. Lastly, for the HCP-YA cohort, family members were always kept within the same cross-validation fold. Hence, all care was taken to avoid any spurious inflation of prediction accuracies in the out-of-sample testing.

**Figure 2:**
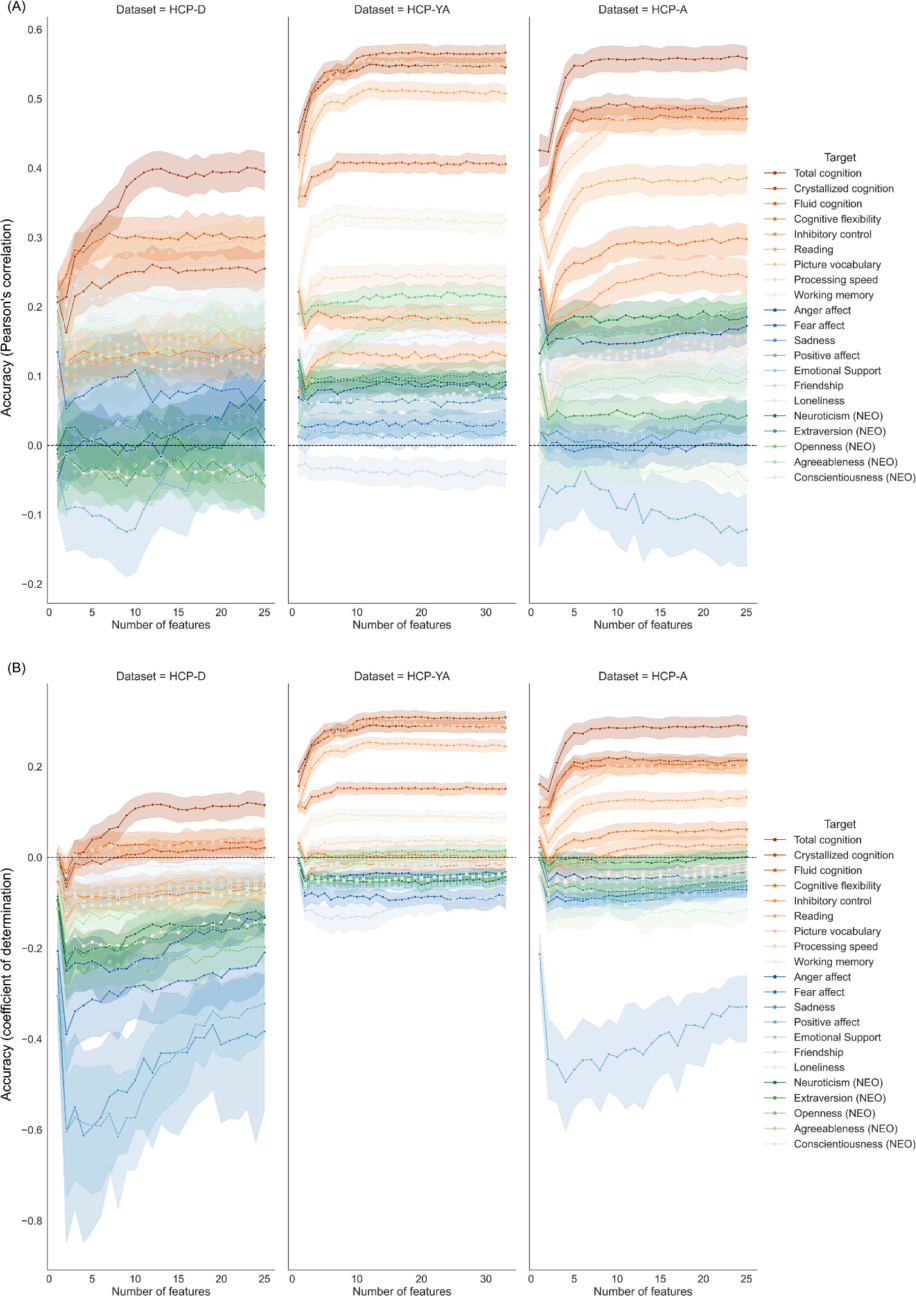
Variation of prediction performance when increasing the number of multimodal neuroimaging feature types integrated, where the accuracies were measured by (A) Pearson’s correlation between predicted and observed psychometric values, and (B) COD score. Each dot in the line plot represents the mean prediction accuracy for one psychometric variable while integrating a certain number of top feature types. Different colours represent different targets of prediction, with orange hues representing targets from the cognition domain, blue hues representing targets from the emotion domain, and green hues representing targets from the personality domain.

Overall, cognition variables were better predicted compared to emotion or personality variables, and they were generally better predicted in HCP-YA and HCP-A than in HCP-D. This can be seen in Fig. 2 that shows the variation of behaviour prediction performance with increasing number of feature types included in integrated-features set models, representing the level of multimodal integration of the models. For these better predicted psychometric variables, rather substantial improvements in prediction performance were observed, but only with a small number of (usually fewer than 10) integrated feature types. Among psychometric variables in the emotion and personality domains, improvement in prediction performance was often not observed when integrating multimodal features. Most trends observed are similar across the two metrics of prediction performance, with the coefficient of determination (COD) score being the stricter measure.

In HCP-D, the best predicted psychometric variable is total cognition, with correlation accuracies reaching R = 0.40 and COD accuracies reaching R2 = 0.12. This best accuracy was achieved by integrating 23 feature types. In HCP-YA, the best predicted psychometric variables are crystallized cognition, total cognition, and picture vocabulary, with correlation accuracies reaching R = 0.57, 0.55, and 0.56, as well as COD accuracies reaching R2 = 0.31, 0.29, 0.30. These accuracies were achieved by integrating 19, 12 (for correlation accuracy) to 13 (for COD accuracy), and 12 feature types respectively. In HCP-A, the best predicted psychometric variable is total cognition, with correlation accuracies reaching R = 0.56 and COD accuracies reaching R2 = 0.29. This best accuracy was achieved by integrating 24 feature types.

### Saturated Prediction Performance with Small Sets of Features.

**Figure 3:**
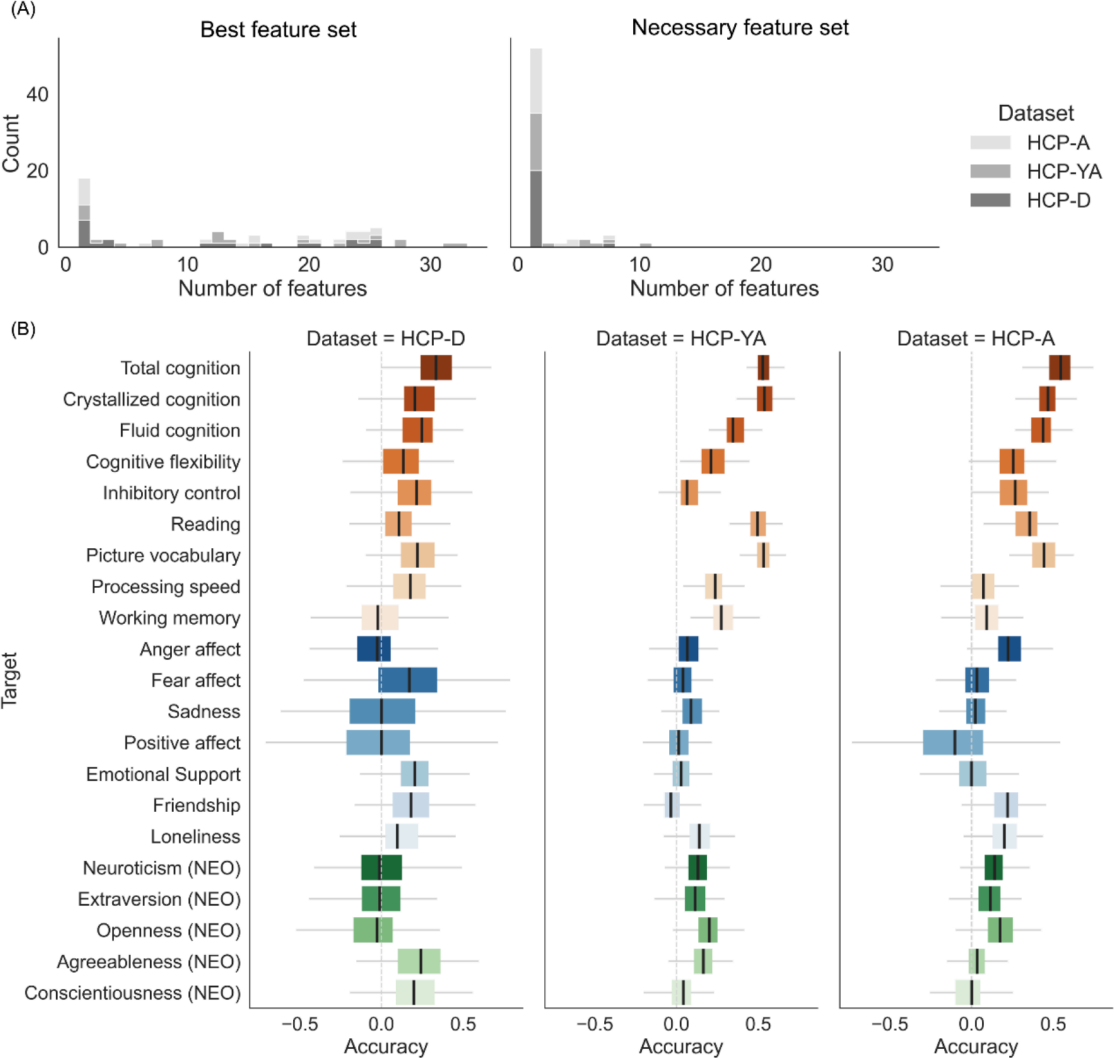
(A) Distribution of number of feature types integrated for the “best” feature sets (left column) and for the “necessary” feature sets (right column) based on correlation accuracy. Colour hues represent which dataset the feature sets were found in. (B) Prediction accuracies by correlation for each psychometric variable using the integrated-features set model corresponding the “necessary” feature set, in each cohort. The light grey vertical line in each column marks the accuracy of R = 0.

For the prediction of each psychometric variable in each cohort, we defined the “best” feature set, corresponding to the integrated-features set model with the best numerical prediction accuracy, as well as the “necessary” feature set, corresponding to the integrated-features set model with the fewest number of integrated feature types that still showed statistically comparable prediction accuracy with the “best” model. Figure 3A shows the distribution of number of integrated feature types across psychometric variables and cohorts for the “best” and “necessary” feature sets, determined based on correlation accuracy. While the number of feature types included in the “best” feature set is roughly uniformly distributed from 1 to more than 30, the number of feature types for the “necessary” feature set is mostly 1 and never more than 10. In other words, at most 10 feature types are needed for multimodal integration before prediction performance saturates. Similar distributions were observed for the feature sets determined based on COD accuracy exhibited similar distributions (Figure S1A).

Figure 3B and S1B show the prediction accuracies for the integrated-features set models corresponding to the “necessary” feature set for each psychometric variable in each cohort, for correlation accuracy and COD accuracy respectively. Compared to the numerically best case, the total cognition prediction in HCP-D achieved correlation accuracies of R = 0.34 and COD accuracies of R^2^ = 0.04, using 6 feature types for multimodal integration. For HCP-YA, the predictions of crystallized cognition, total cognition, and picture vocabulary achieved correlation accuracies of R = 0.54, 0.53, and 0.53, and COD accuracies of R^2^ = 0.28, 0.27, and 0.27, using 5, 6, and 7 feature types for multimodal integration respectively. In HCP-A, the total cognition prediction achieved correlation accuracies of R = 0.53 and COD accuracies of R^2^ = 0.25 while using only 4 feature types in multimodal integration. The differences in prediction accuracies between the integrated-features set model corresponding to the “best” and the “necessary” feature sets are small for well predicted psychometric variables, but can be more substantial when the “best” accuracy was already low.

### Useful Feature Types.

**Figure 4:**
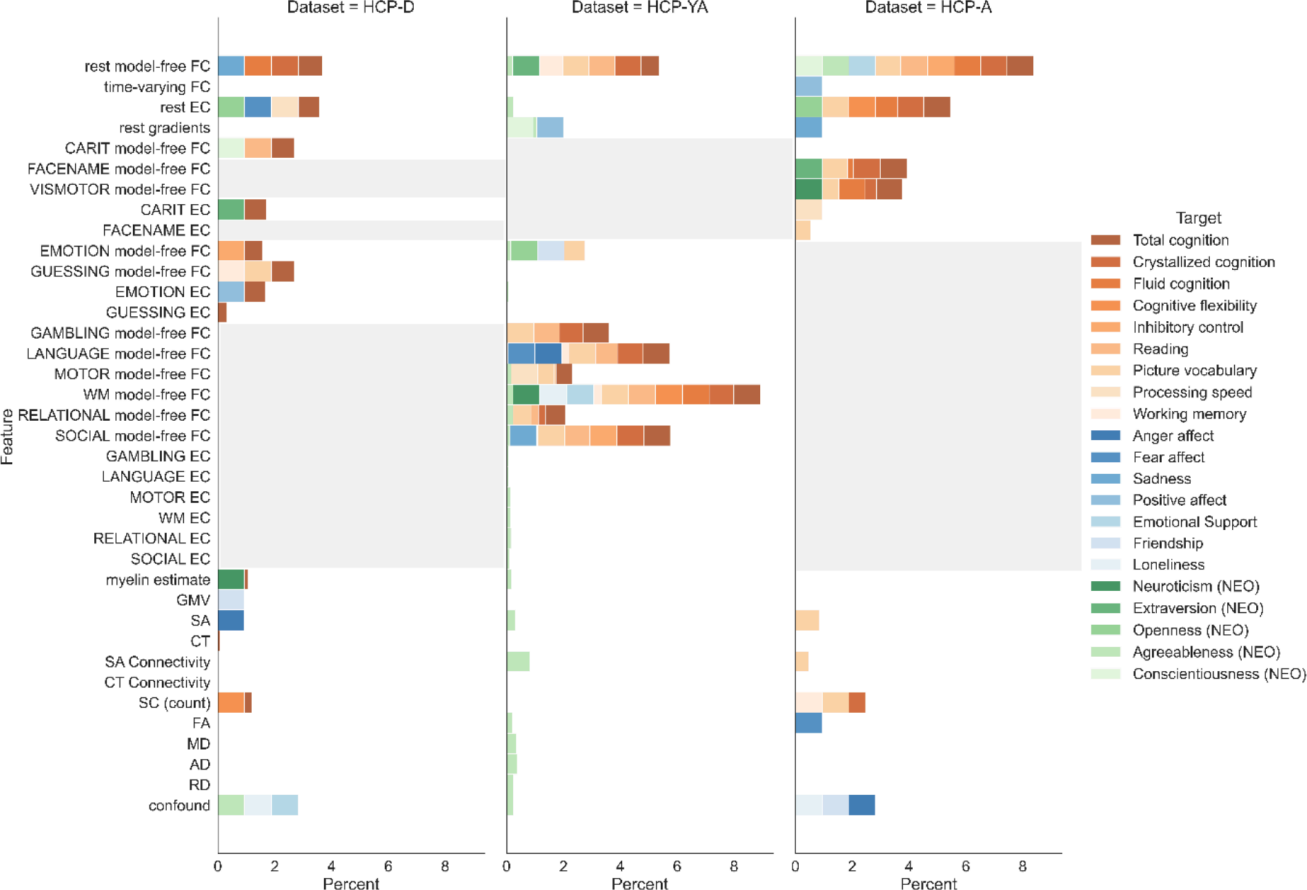
Frequency of each feature type being part of the “necessary” feature set for behaviour prediction in each dataset (left column: HCP-D; middle column: HCP-YA; right column: HCP-A), based on both correlation and COD accuracies. Colours represent different targets of prediction, with orange hues representing targets from the cognition domain, blue hues representing targets from the emotion domain, and green hues representing targets from the personality domain. The light grey area in each column represents the t-fMRI features unavailable in the corresponding cohort.

To identify which types of neuroimaging features are useful for behaviour prediction, we examined the composition of the “necessary” feature sets. Figure 4 shows the frequency of each feature type being useful for reaching the saturated prediction performance across predictions of all psychometric variables in each cohort. Overall, model-free FC from resting-state and most task conditions can be considered useful in all three cohorts. In the developmental and aging cohorts, resting-state effective connectivity, streamline count based structural connectivity, and confounding variables could also be useful for the prediction of multiple behaviour variables. Regarding the non-brain features, the confounding variables were only useful for the predictions of some mental health and personality phenotypes that were less well predicted, such as emotional support, loneliness, and agreeableness. However, the usefulness of each feature type varies across behavioural domains and cohorts.

In HCP-D, other useful feature types include resting-state effective connectivity, myelin content estimate, gray matter volume, cortical surface area, cortical thickness, streamline count based structural connectome, and confounding variables. In HCP-YA, only the predictions of positive affect and personality measures involve non-FC feature types, such as resting-state gradient loadings, cortical surface area, cortical surface area based connectivity, etc. In HCP-A, the prediction of cognition variables also depends on effective connectivity from resting-state, the Go/NoGo task, and the FaceName task, cortical surface area, cortical surface area based connectivity, and streamline count based structural connectome. Similarly, the prediction of openness involves resting-state effective connectivity. The prediction of emotion measures requires very different types of features, including time-varying FC, resting-state gradient loadings, fractional anisotropy, and confounding variables.

**Figure 5:**
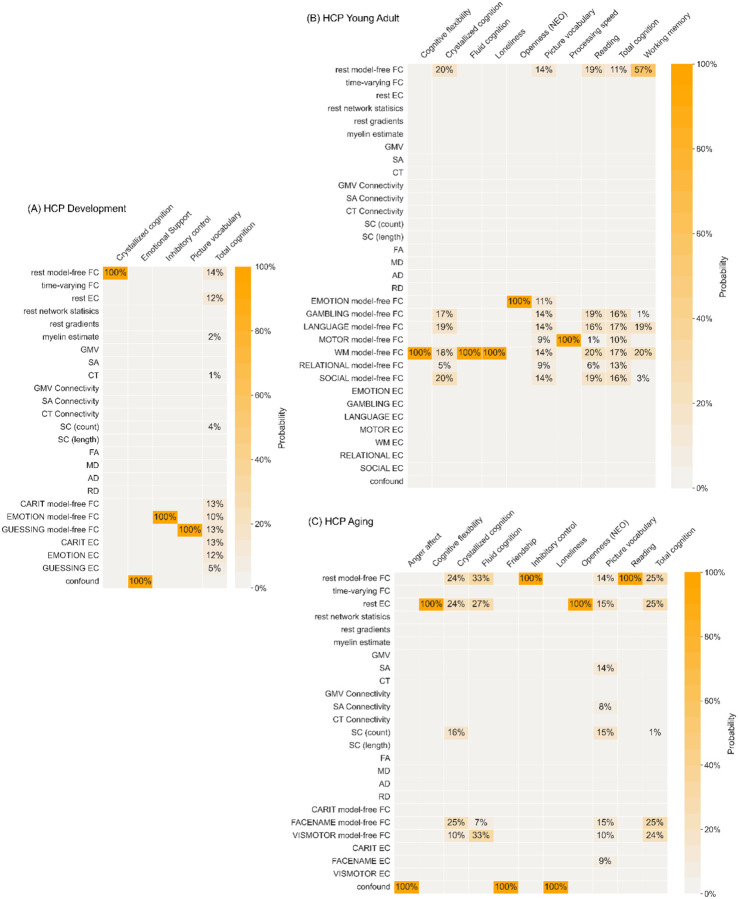
Frequency of each feature type being useful for the prediction of psychometric variables with COD accuracies of R^2^ > 0, in (A) HCP-D, (B) HCP-YA, and (C) HCP-A. The colour scale represents the percentage frequency of a feature type being part of the “necessary” feature set for a psychometric variable in a cohort, with more saturated colours representing higher frequency. The percentage frequency is based on both correlation and COD accuracies.

As the predictions of many psychometric variables reached only low accuracies, a more informative investigation of useful neuroimaging feature types may mean examining only “valid” predictive models. Due to the large number of predictive models implemented in our analysis, we applied a simple threshold to focus on predictions where the stricter COD accuracy reaches R^2^ > 0, where predictors may be assumed to useful features. Figure 5 shows the frequency of each feature type being useful only for predictions of psychometric variables satisfying this threshold in each cohort. Two trends emerge immediately across all three cohorts. First, the composite score of total cognition in all cohorts, and, in some cohorts, the composite scores of crystallized cognition and fluid cognition, picture vocabulary, and reading require multiple different types of features to reach saturated prediction performance. Second, prediction of psychometric variables like cognitive flexibility, inhibitory control, loneliness, and openness always reached saturated performance with a single feature type, i.e., without integrating multimodal information.

The types of features involved in these trends differ, however, across cohorts. In HCP-D, the multimodal integration uses mostly resting-state model-free FC, resting-state effective connectivity, all task model-free FC, and all task effective connectivity, as well as less frequently myelin content estimate, cortical thickness, and streamline count based structural connectome. For psychometric variables requiring no multimodal integration, the useful single feature types include resting-state model-free FC, Emotion model-free FC, Guessing model-free FC, and confounding variables. In HCP-YA, the useful features are always the model-free FC from resting-state and different task conditions. In HCP-A, the multimodal neuroimaging features integrated mostly include resting-state model-free FC, resting-state effective connectivity, cortical surface area, cortical surface area based connectivity, streamline count based structural connectome, FaceName model-free FC, Visuomotor model-free FC, and FaceName effective connectivity. On the other hand, the feature types for reaching saturated performance without multimodal integration include resting-state model-free FC, resting-state effective connectivity, and confounding variables.

## Discussion

We systematically evaluated multimodal brain-based behaviour prediction across a wide range of behavioural variables and neuroimaging features, in three large cohorts covering different age ranges. Generally, performance of predictive models saturated by integrating a few types of neuroimaging features, mostly involving model-free FC from rs-fMRI and t-fMRI. Further analysis of only “valid” predictive models revealed that while the predictions of some psychometric variables require a range of features and often from all imaging modalities, predictions of other psychometric variables were dominated by single feature types. To summarise, the need for multimodal information integration and the type of features or modalities required for brain-based behaviour prediction are dependent on the behavioural target and the studied population.

Examining the trend of prediction performance further, it is worth noting that the performance achieved for each psychometric variable was dependent not only on the level of multimodal integration, but also on the domain of the psychometric variable and on the cohort. Similar to previous reports, our results demonstrated that predictions of cognition measures are generally better than predictions of emotion or personality measures (Li et al., 2019; Mansour et al., 2021; Wu et al., 2021; He et al., 2022; Chen et al., 2022; Ooi et al., 2022; Zhi et al., 2024; Rauland et al., 2025). This domain effect may be due to either the worse reliability of self-reported measures in emotion and personality domains, or the lack of variance related to mental health in healthy cohorts (Uher, 2015; Dubois et al., 2018; Dhamala et al., 2023; Wu et al., 2023; Gell et al., 2024). Furthermore, we observed that composite scores encompassing multiple cognitive functions are better predicted than individual cognitive task scores (Dhamala et al., 2022). Generally, prediction accuracies were lower in HCP-D compared to the other two cohorts. Similar drops in prediction accuracies from R ~ 0.5 to R ~ 0.4 for the prediction of total cognition or similar measures in developmental subjects was found in previous studies (Ooi et al., 2022; Tetereva et al., 2025). These low prediction accuracies in the HCP-D cohort may be related to the general greater movement and lower imaging quality in developmental subjects (Somerville et al., 2018), and the standardised image processing designed mainly based on adult data. Furthermore, prediction performance of each psychometric variable also varies to different extents across all three cohorts, demonstrating complex interactions of brain-behaviour relationships with age groups, imaging protocols, and other cohort idiosyncrasies.

Focusing on the “valid” predictive models, a clear differentiation was observed between psychometric variables where a single feature type dominated the prediction and those where multimodal integration was required. The predictive models requiring multimodal integration only involved the three composite cognition scores, picture vocabulary, and reading, also corresponded to the top prediction performance in each cohort. Our results suggest that multimodal integration is most likely to benefit behaviour prediction when the target is a multifaceted score relating to multiple cognitive functions, or for targets measuring high-level integrative functions such as language skills. Furthermore, our results suggest that it may be necessary to integrate features from all four modalities investigated, i.e., rs-fMRI, t-fMRI, sMRI, and dMRI, in order to predict multifaceted cognition variables, although the modalities useful in multimodal integration still differ across cohorts. In particular, s-MRI and d-MRI features received more importance in the aging cohort compared to the others, likely due to relevant interindividual differences in brain structural degeneration. Furthermore, this reliance on both structural and functional features corroborates findings from other studies suggesting that age-related changes in cognition are related to multimodal brain markers (Bennett and Madden, 2014; Hedden et al., 2016). Thus, a better understanding in interindividual variability in cognitive aging likely requires a multimodal view at the brain level.

In contrast, the single dominating feature types mostly correspond to predictive models with relatively lower prediction accuracies in each cohort. It is still worth noting that the useful feature types vary greatly across psychometric variables and ever more across cohorts. In HCP-YA, the single dominating feature types were always t-fMRI model-free FC, with the working memory task (WM) being most often the useful feature type. The close relation between the working memory task with cognitive ability has also been reflected in behaviour prediction in other studies (Pat et al., 2022; Ooi et al., 2022; Tetereva et al., 2025). In HCP-A, more importance was observed for resting-state model-free FC and resting-state effective connectivity, possibly due to the lack of strongly relevant task conditions that provide complementary information to resting-state features. In HCP-A and HCP-D, confounding variables were also found to dominate prediction of anger affect, friendship, loneliness, and emotional support. As these psychometric variables tend to be socially determined, the interindividual differences for these behaviours may be more correlated with sociodemographic factors like age and gender. Nonetheless, the usefulness of sociodemographic factors for behaviour prediction is limited by the lower prediction accuracies obtainable for these psychometric variables from the emotion domain.

The role of rs-fMRI and t-fMRI features and the approach of combining these features in behaviour prediction has been an important topic of discussion in the field (Burr et al., 2019; Avery et al., 2020; Jiang et al., 2020b; Ooi et al., 2022; Pat et al., 2022; Tetereva et al., 2025). In particular, t-fMRI features from cognition-related task conditions, such as language and working memory, have been found to be the most predictive for multiple behavioural variables (Greene et al., 2018; Jiang et al., 2020a; Ooi et al., 2022; Pat et al., 2022; Tetereva et al., 2025; also see Fig. 5B). These findings bring forth new challenges for brain-based behaviour prediction in the use of t-fMRI modality, as t-fMRI collection often differ across datasets, motivated by differences in research questions investigated and/or limitations of certain age and clinical groups (Alfaro-Almagro et al., 2018; Casey et al., 2018; Harms et al., 2018). On the other hand, the high relevance of t-fMRI in relation to brain functions makes t-fMRI features useful for investigating specifically targeted questions such as the discovery of neurobiomarker for sustained attention (Rosenberg et al., 2016; Rosenberg et al., 2018; Rosenberg et al., 2020). In our analysis, the “Guessing” task measuring reward processing in HCP-D was also found to be useful for the prediction of picture vocabulary, possibly due to the relevance of reward processing in developing crystallised cognition (Baldassarre, 2011; Murayama and Elliot, 2011; Vollmeyer and Rheinberg, 2013; Yan and Davison, 2013; Oudeyer et al., 2016). This points to the different relevance of t-fMRI for understanding interindividual variability in behaviour across different age stages, specifically for cognitive processes matured during child development or cognitive abilities that may decline during aging (Mooraj et al., 2025).

Adding to existing literature, our analysis demonstrated the importance of effective connectivity features, in particular the rDCM-based effective connectivity in developmental and aging cohorts. Many studies have used DCM-based effective connectivity to investigate brain-behaviour relationships, examining aspects such as cognitive control, working memory, and cognitive flexibility (Mechelli et al., 2003; Rowe et al., 2008; Witt and Stevens, 2013; Nee and D’Esposito, 2017; Jung et al., 2018; Qiao et al., 2020). Nevertheless, most existing studies made use of DCM with low number of nodes, usually using fewer than 10 nodes (Witt and Stevens, 2013; Nee and D’Esposito, 2017; Jung et al., 2018; Qian et al., 2020). Our results demonstrated the potential of effectivity connectivity based on fine atlas with hundreds of nodes in studying brain behaviour relationships. Moving beyond purely model-driven, our results suggest that effective connectivity may also be useful in combination with model-free FC features. Furthermore, resting-state effective connectivity was useful for the prediction of a range of cognition variables, as well as openness, in the aging cohort. These prediction models may have capitalised on the aging-induced differences in brain connectivity which are directional and correlated to cognitive variability, for instance in cognitive control or working memory (Archer et al., 2016; Tsvetanov et al., 2016; Chand et al., 2017; Heinzel et al., 2017). In line with past literature, our study suggests that neuroimaging features assessing directional connectivity can contribute to a better understanding of cognitive aging.

In this study, we selected commonly used psychometric variables, neuroimaging features and model design. Nevertheless, many differences with other studies remain that may have caused divergence in conclusions drawn. To start, the variable of general cognition or intelligence is one of the best predicted behavioural variables in our analysis and a widely studied target of brain-based prediction (Sui et al., 2020; Vieira et al., 2022; Yeung et al., 2022). The prediction of general cognition or intelligence may make use of intelligence test scores (Jiang et al., 2020a; Tetereva et al., 2025), composite scores from multiple tests (Dhalama et al., 2021; Rasero et al., 2021; Tetereva et al., 2025), or derived factor scores (Mansour et al., 2021; Ooi et al., 2022). These representations of general cognition vary in levels of complexity and range of cognitive functions involved, and thus may require different level of multimodal integration (Rasero et al., 2021; Ooi et al., 2022). Many neuroimaging features have not been considered in our analysis due to their less common usage, although they have been reported to be predictive. Other potentially useful neuroimaging features may include t-fMRI contrasts (Pat et al., 2022; Teterevan et al., 2025), time-varying FC and gradient features computed using different methods (Przezdzik et al., 2019; Hong et al., 2020; Dahan et al., 2021; Ikeda et al., 2022), and features based on structure-function coupling (Suares et al., 2020). Moreover, difference in the choice of atlases and granularity of the atlas may lead to different outcomes. Our choice of granularity was motivated by the conclusions from previous work that 200–300 parcels represent an optimal range for surface data (Wu et al., 2021). Nevertheless, we have not considered whether the same granularity would be optimal for different MRI modalities and feature types. In addition, vertex-wise and voxel-wise grey matter volume features have been shown to dominate the prediction of visual working memory (Xiao et al., 2021), which we have not been able to include in the current framework due to overfitting concerns. Finally, the impacts of different machine learning algorithms and cross-validation implementations have not been thoroughly investigated, although studies have reported no vast difference in prediction performance using different machine learning algorithms such as elastic net, support vector regression, linear ridge regression, and kernel ridge regression (Henneghan et al., 2020; Wu et al., 2021; Li et al., 2021; Xiao et al., 2021; Ooi et al., 2022). In general, the methodological decisions in this study were made to cover most common scenarios, but cannot reflect the requirements of more specific aims.

To conclude, we investigated systematically the benefits of using multimodal neuroimaging features in behaviour prediction, across cohorts covering multiple age groups and an extensive range of psychometric variables and neuroimaging features. We found that the need for multimodal data in behaviour prediction varies depending on the cohort and the type of psychometric variable, with multifaceted cognition requiring more features integration. Nevertheless, for such complex variables, no more than ten types of features are required for maximising prediction performance. For less complex scores, single feature types such as resting-state and task model-free FC tend to suffice for prediction. Mostly, rs-fMRI and t-fMRI features are useful for behaviour prediction, while sMRI and dMRI features are occasionally required for multimodal integration too. Overall, studies in developmental and aging populations are more likely to benefit from considering alternatives features to model-free connectivity in order to better capture brain interindividual variability for predictive modelling and likely to better understand interindividual variability in cognitive development and aging. Finally, we caution that while the multimodal integration approach proves useful in many cases, the lack of resources for cross-cohort generalizability assessment of the t-fMRI data remains as an important challenge to truly embrace the perspective of task-fMRI for predictive modelling and biomarker development.

## Methods

We made use of data from 3 large cohorts, HCP-YA (N = 796), HCP-A (N = 700), and HCP-D (N = 593). For each dataset, 25–33 types of neuroimaging features were extracted from 4 imaging modalities (rs-fMRI, t-fMRI, sMRI, and dMRI), and 21 psychometric variables were selected as prediction targets from 3 domains (cognition, emotion, and personality). Prediction models were trained and tested using a nested cross-validation scheme, where hyperparameters were estimated using the inner training data. A stacking procedure was implemented to integrate different neuroimaging features. To avoid data and information leakage, any parameter required for neuroimaging features based on a group model (e.g., for functional gradient) was computed only using training subjects and applied to the test set in each cross-validation split. Furthermore, all input features to the “stacked” (i.e. meta-level) model were also computed in a nested cross-validation to prevent leakage. Lastly, for the HCP-YA cohort, family members were always kept within the same cross-validation fold. Hence, all care was taken to avoid any spurious inflation of prediction accuracies in the out-of-sample testing.

### Data and Preprocessing.

We used phenotype and imaging data from the latest releases of the three datasets, the HCP-YA S1200 Release and the HCP-Lifespan 2.0 Release of HCP-A and HCP-D (see a summary of demographic and imaging information in Table 1). The HCP-YA cohort includes healthy young adults from families with twins and non-twin siblings. As an extension, the HCP Lifespan cohorts include healthy older adults in HCP-A and healthy younger developmental participants in HCP-D. Imaging data from all modalities for HCP-YA and from all modalities except dMRI for HCP-A and HCP-D were preprocessed using the HCP Minimal Processing Pipelines (Glasser et al., 2013). For each cohort, we only considered subjects with imaging data from every modality and all selected psychometric variables.

For the rs-fMRI and t-fMRI modalities, we used the ICA-FIX (Smith et al., 2013; Griffanti et al., 2014; Salimi-Khorshidi et al., 2014) denoised data on the fsLR surface (aligned by the MSMAll pipeline) and in the MNI152 volumetric space. Nuisance regressors including white matter signals, cerebrospinal fluid signals, global signals, and their derivatives were extracted from the volumetric data and regressed out from both surface and volumetric data.

For sMRI, we used the preprocessed T1-weighted volumes in the MNI152 space, the estimated myelin maps (Glasser and Van Essen, 2011) on the fsLR surface and in subject-wise T1-weighted space, as well as the output from the FreeSurfer “recon-all” pipeline (Fischl, 2012).

For dMRI data in HCP-A and HCP-D, we preprocessed the raw data provided using a Python implementation of the diffusion preprocessing pipeline from the HCP Pipelines (Glasser et al., 2013), using default parameters so that the processing was as close to that in HCP-YA as possible. Probabilistic tractography was performed using the preprocessed diffusion data for all three datasets using the “iFOD2” algorithm from MRTrix3 (Tournier et al., 2010; Smith et al., 2012; Smith et al., 2015; Tournier et al., 2019). Default values were used for most parameters, except “maxlength”, “max_attempts_per_seed”, and “select”, where we used the recommended settings for the Schaefer atlas (Schaefer et al., 2018) from a previous study (Jung et al., 2021). Diffusion tensor imaging (DTI) feature maps of fractional anisotropy (FA), mean diffusivity (MD), axial diffusivity (AD), and radial diffusivity (RD) were also generated based on the preprocessed diffusion data using the “dtifit” tool from FSL (Jenkinson et al., 2012). These maps were then mapped onto skeletons using the Tract-Based Spatial Statistics (TBSS) pipeline from FSL (Jenkinson et al., 2012).

### Multimodal neuroimaging features.

A range of neuroimaging features were considered for each MRI modality, in order to cover most commonly used feature types in the field (see a summary of descriptions in Table 2). For all rs-fMRI, t-fMRI, sMRI, and structural connectome features, data were parcellated using the 300-parcel Schaefer cortical atlas (Schaefer et al., 2018) and the matching 50-parcel Melbourne subcortex atlas (Tian et al., 2020). For the diffusion tensor model (DTI) features, the JHU DTI-based white matter atlas (Mori et al., 2005; Wakana et al., 2007; Hua et al., 2008) was used for parcellation.

For resting-state and every task condition, the region-wise mean timeseries were computed and concatenated across runs. Model-free functional connectivity (FC) was computed for each condition as the Pearson’s correlation between pairs of region-wise concatenated timeseries. The upper triangular portions of the FC matrices were extracted and Fisher’s z transformed. Using these resting-state timeseries, time-varying FC was also estimated as the first-order autoregressive model parameters (Liégeois et al., 2019). While this is more commonly called dynamic FC, the term “time-varying” is used here instead to avoid confusion with dynamic causal modelling based features. Based on the computed resting-state model-free FC, we further computed network statistics, including characteristic path length, global efficiency, modularity, and region-wise participation coefficients, using the brain connectivity toolbox (Rubinov, et al., 2009; Rubinov and Sporns, 2010), as well as gradient loadings using diffusion mapping from the mapalign package (https://github.com/sensein/mapalign; Coifman and Lafon, 2006; Langs et al., 2015; Margulies et al., 2016).

Using the provided T1-weighted to T2-weighted intensity ratio maps, we extracted region-wise myelin estimate by averaging the myelin content estimated by T1-to-T2 ratio (Glasser and Van Essen 2011) within each brain region. Cortical surface area (SA) and cortical thickness (CT) values were obtained with the tables from the “mris_anatomical_stats” command from FreeSurfer (Fischl, 2012). Gray matter volume (GMV) values were extracted from the T1-weighted volumes using the “mri_segstats” command from FreeSurfer (Fischl, 2012). To adjust for brain size, cortical surface area values were divided by the intracranial volumes (ICV) to the power of 2/3, while gray matter volume values were divided by ICVs, and cortical thickness values were divided by the mean across the cortex. For each type of morphometry measure, a connectivity matrix was estimated using structural co-registration (sCoRe; https://github.com/katielavigne/score), representing the extent of covariation between pairs of brain regions in terms of a morphometry measure.

With the tractograms estimated from the dMRI data, we computed structural connectome (SC) using the “tck2connectome” function from MRTrix3 (Smith et al., 2015; Tournier et al., 2019). Two structural connectome matrices were computed for each subject, one measuring the number of streamlines between pairs of brain regions, and one where the streamline counts were weighted by the length of the streamlines. Using the skeletonised DTI feature maps, we extracted the region-wise DTI features with the JHU white matter atlas.

Finally, we estimated effective connectivity (EC) for resting-state and all task conditions using a Python implementation of the regression dynamic causal modelling (rDCM) toolbox (Marreiros et al., 2008; Stephan et al., 2008; Frässle et al., 2017; Frässle et al., 2018; Frässle et al., 2021). For all conditions, the streamline count based structure connectome was used as the prior for estimation. For all task conditions, the driving inputs were modelled as an array of ones during event blocks and zeros elsewhere.

### Psychometric Variables.

We considered 21 psychometric variables that are available in all three cohorts, covering behavioural domains of cognition, emotion, and personality (see a summary of descriptions in Table 3; see Table S1 for the original variable names in each dataset). For cognition and emotion domains, we selected psychometric variables representing distinct behavioural functions or traits, based on the same or similar tests from the NIH Toolbox across the three cohorts. For the personality domain, we used the five factor measures from the Neuroticism/Extroversion/Openness Five Factor Inventory (NEO-FFI; McCrae and Costa, 2004).

### Feature-wise Models.

Feature-wise models were built separately for each feature types. For rs-fMRI, sMRI, dMRI features, this means that one feature-wise model was built for each feature type, e.g., rest model-free FC model, rest effective connectivity model, gray matter volume model, streamline count based structural connectome model. For t-fMRI features, one feature-wise model was built for each feature type from each task condition, e.g., CARIT model-free FC model, CARIT EC model, FACENAME model-free FC model, FACENAME effective connectivity model. Therefore, in total, 25 feature-wise models were computed for each psychometric variable in HCP-D and HCP-A, while 33 feature-wise models were computed for each psychometric variable in HCP-YA where more task conditions were collected for t-fMRI.

We used the widely used elastic net algorithm for behaviour prediction using confounding variables (Zou and Hasti, 2005), which has often been shown to achieve comparable if not better prediction performance than other linear or even nonlinear algorithms (Wu et al., 2020; Ooi et al., 2022; Tetereva et al., 2025). The feature-wise model was trained and tested with 10 repeats of 10-fold cross-validation. To account for the family structure within the HCP-YA cohort, family members were always kept within the same fold. The hyperparameters (L1 ratio and alpha) were determined via nested 5-fold cross-validation. Prediction accuracy was measured in two ways. First, the correlation accuracy was assessed by computing the Pearson’s correlation between predicted and observed psychometric values. Second, COD accuracy was computed using the “score” function from scikit-learn. For the morphometry-based connectivity features and the gradient loadings feature, estimation of parameters and embeddings respectively was performed only on the training set of subjects and applied to the test set of subjects in each cross-validation fold, in order to avoid data leakage.

As several confounding factors are often thought to contribute substantially to brain-behaviour relationships investigated in brain-based behaviour prediction studies, we seek to explicitly examine their contributions to prediction performance in comparison to the neuroimaging features. In other words, we modelled confounding variables as both features in a confound model, and as confounds in feature-wise models. For all three cohorts, we included age, age^2^, gender, age × gender, age^2^ × gender, handedness, brain size, and ICV as confounding variables. Gender was coded as 1 for female and 2 for male. For HCP-A and HCP-D, brain size and ICV were extracted from the FreeSurfer output file “aseg.stats”.

For the feature-wise models, the eight confounding variables were regressed out during cross-validation, such that the regression coefficients were estimated only using the training set of subjects. On the other hand, the confound model was implemented exactly like feature-like models but using the confounding variables as features without confound regression.

### Integrated-features Set Models.

A stacking procedure was implemented to integrate features from multimodal neuroimaging data and confounding variables. The integrated-features set models (i.e., the “stacked” models) receive prediction output from the feature-wise models and the confound model, which were then used as features for behaviour prediction. More precisely, in each cross-validation fold, the feature-wise models and the confound model computed a predicted psychometric value for each subject based on the same cross-validation split. For subjects in the test split, the values were predicted from a model trained on the subjects in the training split to avoid data leakage. For subjects in the training split, to avoid leakage, the values were predicted in a nested 5-fold cross-validation, where the value for each subject was computed when that subject was part of the inner test split.

The integrated-features set models were then implemented using the Random Forest regression algorithm (Breiman, 2001), which is favoured at the stacking (i.e. meta) level for its robustness and its ability to capture nonlinear interaction effects (Engemann et al., 2020; Pat et al., 2022). The split quality was computed by mean squared error with Friedman’s improvement score. The hyperparameters (number of trees, minimum depth of trees, and number of features to consider when looking for best split) were determined by grid search in a nested 5-fold cross-validation. Prediction accuracy was again measured by Pearson’s correlation between predicted and observed psychometric values and the COD scores.

In order to examine the trend of prediction performance variation by the level of multimodal integration, we implemented multiple integrated-features set models corresponding to different levels of multimodal integration. In each cross-validation fold, the ranking of feature types was assessed based on accuracies in the training split, to avoid leaking knowledge from the test subjects. Starting from the top 2 feature types, integrated-features set models were built using features only from the feature-wise or confound models from these top 2 feature types. The feature space was then expanded to top 3 feature types, top 4 feature types, and so on, until all feature types were included. In this way, we obtained a series of integrated-features set models corresponding to multimodal integration level 2 to level 25 in HCP-D and HCP-A, and to level 33 in HCP-YA.

### Necessary Feature Set.

By counting the feature-wise or confound model of the top feature in each cross-validation fold as the model for multimodal integration level 1, we can form a complete series of prediction models with varying multimodal integration levels. As all models were implemented using the same cross-validation split, their prediction accuracies can be statistically compared by the corrected resampled t test. We thus computed the p value of differences in mean between each pair of models in this series of varying multimodal integration levels, corrected for multiple comparisons (Benjamini and Hochberg, 1995).

To determine which feature types were essential for the prediction of each psychometric variable in each cohort, we first found the model with the best numerical prediction accuracy in each case. The feature types included in this model correspond to the “best” feature set. We then reduced the level of multimodal integration from that of the “best” model and looked for the model with the lowest level of multimodal integration that still showed statistically comparable prediction performance with the “best” model. We consider this model as exhibiting the saturated prediction performance from increasing multimodal integration levels. The feature types included in this model thus correspond to the “necessary” feature set, as they were necessary to reach the saturated level of prediction performance.

## Supplementary Material

Supplementary Files

This is a list of supplementary files associated with this preprint. Click to download.

- SupplementalMaterials.docx

## Figures and Tables

**Table 1.** Demographic and imaging information of datasets used

	HCP-D (Somerville et al., 2018)	HCP-YA (Van Essen et al., 2013)	HCP-A (Bookheimer et al., 2019)
**Number of subjects**	593	796	700
**Age**	14.72±3.88 (8–22 years old)	28.62±3.78 (22–37 years old)	60.16±15.49 (36–100 years old)
**Gender**	318 female, 275 male	448 female, 348 male	392 female, 308 male
**rs-fMRI scans**	4 runs of 6.5 min / 478 frames	4 runs of 14.4 min / 1200 frames	4 runs of 6.5 min / 488 frames
**t-fMRI scans**	2 runs each for Guessing (270 frames), Go/NoGo (CARIT, 290 frames), Emotion (168 frames)	2 runs each for working memory (406 frames), gambling (253 frames), motor (284 frames), language (316 frames), social cognition (274 frames), relational processing (232 frames), emotion processing (176 frames)	1 run each for Visuomotor (VISMOTOR, 184 frames), Go/NoGo (CARIT, 290 frames), FaceName (335 frames)
**fMRI repetition time (TR)**	800 ms	720 ms	800 ms
**fMRI spatial resolution**	2mm isotropic	2mm isotropic	2mm isotropic
**sMRI spatial resolution**	0.8mm isotropic	0.7mm isotropic	0.8mm isotropic
**dMRI number of directions**	98, 99	95, 96, 97	98, 99
**dMRI b values**	1500, 3000	1000, 2000, 3000 (approximately equal number of acquisitions each)	1500, 3000
**dMRI spatial resolution**	1.5mm isotropic	1.25mm isotropic	1.5mm isotropic

**Table 2.** Brief descriptions of neuroimaging features selected

Modality	Feature type	Feature name	Description
**rs-fMRI**	Model-free FC	rest model-free FC	Pearson’s correlation between pairs of region-wise mean timeseries
	Time-varying FC	time-varying FC	First-order autoregressive model parameters between pairs of brain regions (see Liegeois et al., 2019)
	Effective connectivity	rest EC	Effective connectivity between pairs of brain regions estimated using rDCM (Marreiros et al., 2008; Stephan et al., 2008; Frassle et al., 2017; Frassle et al., 2018; Frassle et al., 2021)
	Network statistics	rest network statistics	Characteristic path length, global efficiency, modularity, and region-wise participation coefficient based on model-free FC using BCT (Rubinov, et al., 2009; Rubinov and Sporns, 2010)
	Gradient loadings	rest gradients	Region-wise loading on each gradient component, computed based on model-free FC using diffusion mapping (similar to Margulies et al., 2016).
**t-fMRI**	Model-free FC	task model-free FC (e.g., CARIT model-free FC)	Pearson’s correlation between pairs of region-wise mean timeseries
	EC	task EC (e.g., CARIT EC)	Effective connectivity between pairs of brain regions estimated using rDCM (Marreiros et al., 2008; Stephan et al., 2008; Frassle et al., 2017; Frassle et al., 2018; Frassle et al., 2021)
**sMRI**	Myelin content estimate	myelin estimate	Region-average T1-weighted to T2-weighted intensity ratio (Glasser & Van Essen 2011)
	Morphometry measures	GMV, SA, CT	Region-wise GMV, SA, and CT estimated using FreeSurfer (Fischl, 2012)
	Morphometry based connectivity	GMV Connectivity, SA Connectivity, CT Connectivity	Morphometry-based “anatomical” connectivity using SCoRe (https://github.com/katielavigne/score).
**dMRI**	Structural connectome	SC (count), SC (length)	Number of streamlines (count), and the contribution of streamlines scaled by their lengths (length) between pairs of brain regions using “tck2connectome” from MRTrix3 (Smith et al., 2015; Tournier et al., 2019)
	Tensor-based diffusion measures	FA, MD, AD, RD	Region-wise DTI measures using “dtifit” and “TBSS” from FSL (Jenkinson et al., 2012)

**Table 3.** Brief descriptions of psychometric variables selected

Domain	Psychometric variable	Description
**Cognition**	Total cognition	Age-adjusted composite score based on all cognition measures from the NIH toolbox
	Crystallized cognition	Age-adjusted composite score based on Dimension Change Card Sort, Flanker, Picture Sequence Memory, List Sorting, and Pattern Comparison test scores
	Fluid cognition	Age-adjusted composite score based on Picture Vocabulary and Oral Reading Recognition test scores
	Cognitive flexibility	Age-adjusted Dimension Change Card Sort test score assessing the cognitive flexibility aspect of executive function
	Inhibitory control	Age-adjusted Flanker test score assessing the inhibitory control aspect of executive function
	Reading	Age-adjusted Oral Reading Recognition test score assessing the reading aspect of language skills
	Picture vocabulary	Age-adjusted Picture vocabulary test score assessing the receptive vocabulary aspect of language skills
	Processing speed	Age-adjusted Pattern Comparison test assessing mental processing speed
	Working memory	List Sorting test score assessing working memory
**Emotion**	Anger affect	Negative affect test score assessing irritability, frustration, and efforts to control anger
	Fear affect	Negative affect test score assessing fear, hyperarousal, and somatic symptoms related to arousal
	Sadness	Negative affect test score assessing poor mood and negative perception of the self, the world, and the future
	Positive affect	Positive affect test score assessing feelings of pleasurable engagement with the environment
	Emotional support	Social relationships test score assessing the participant’s perception of the availability of emotion support
	Friendship	Social relationships test score assessing the participant’s perception of the availability of friends or companions
	Loneliness	Social relationships test score assessing perceptions of being alone, lonely, or socially isolated from others
**Personality**	Neuroticism (NEO)	Neuroticism measure of the five-factor personality model from NEO-FFI
	Extraversion (NEO)	Extraversion measure of the five-factor personality model from NEO-FFI
	Openness (NEO)	Openness measure of the five-factor personality model from NEO-FFI
	Agreeableness (NEO)	Agreeableness measure of the five-factor personality model from NEO-FFI
	Conscientiousness (NEO)	Conscientiousness measure of the five-factor personality model from NEO-FFI

## Data Availability

All data were managed via version-controlled DataLad datasets (Halchenko, et al., 2021) that are either publicly available, or were provided by an institutional data management system when public sharing was prevented by the terms of the respective data usage agreements. The HCP-YA imaging data were accessed via the public DataLad dataset provided at https://github.com/datalad-datasets/human-connectome-project-openaccess (2e2a8a70–3eaa-11ea-a9a5-b4969157768c@a33e528) which interfaces the HCP Open Access dataset (https://registry.opendata.aws/hcp-openaccess) on AWS S3. The unrestricted and restricted phenotype data were downloaded from the ConnectomeDB (https://db.humanconnectome.org) after accepting the Open Access Data User Terms and Restricted Access Data Use Terms respectively. The HCP-A and HCP-D imaging and phenotype data were downloaded from the NIMH Data Archive (NDA; https://nda.nih.gov), after applying for the Data Use Certification. The associated study ID is 3146 (http://dx.doi.org/10.15154/w0cr-8r66). All code for feature extraction and prediction are publicly available at https://github.com/jadecci/mpp. Any annexed content in the repository can be accessed at https://gin.g-node.org/jadecci/MPP. The Python implementation of the HCP Diffusion Pipeline is available at https://github.com/jadecci/hcp_pipeline_diffusion_py. The Python implementation of the regression dynamic causal modelling toolbox is available at https://github.com/jadecci/rDCM_py.
